# Supplementary material for: Identification of a Fatty Acid for Diagnosing Non-Alcoholic Steatohepatitis in Patients with Severe Obesity Undergoing Metabolic Surgery
Source: Biomedicines. 2022 Nov 14;10(11):2920. doi: 10.3390/biomedicines10112920 (PMC9687903; doi:10.3390/biomedicines10112920)
Supplement: Supplementary file 1 [file biomedicines-2025590-SI.pdf › biomedicines-2025590-SI.pdf]

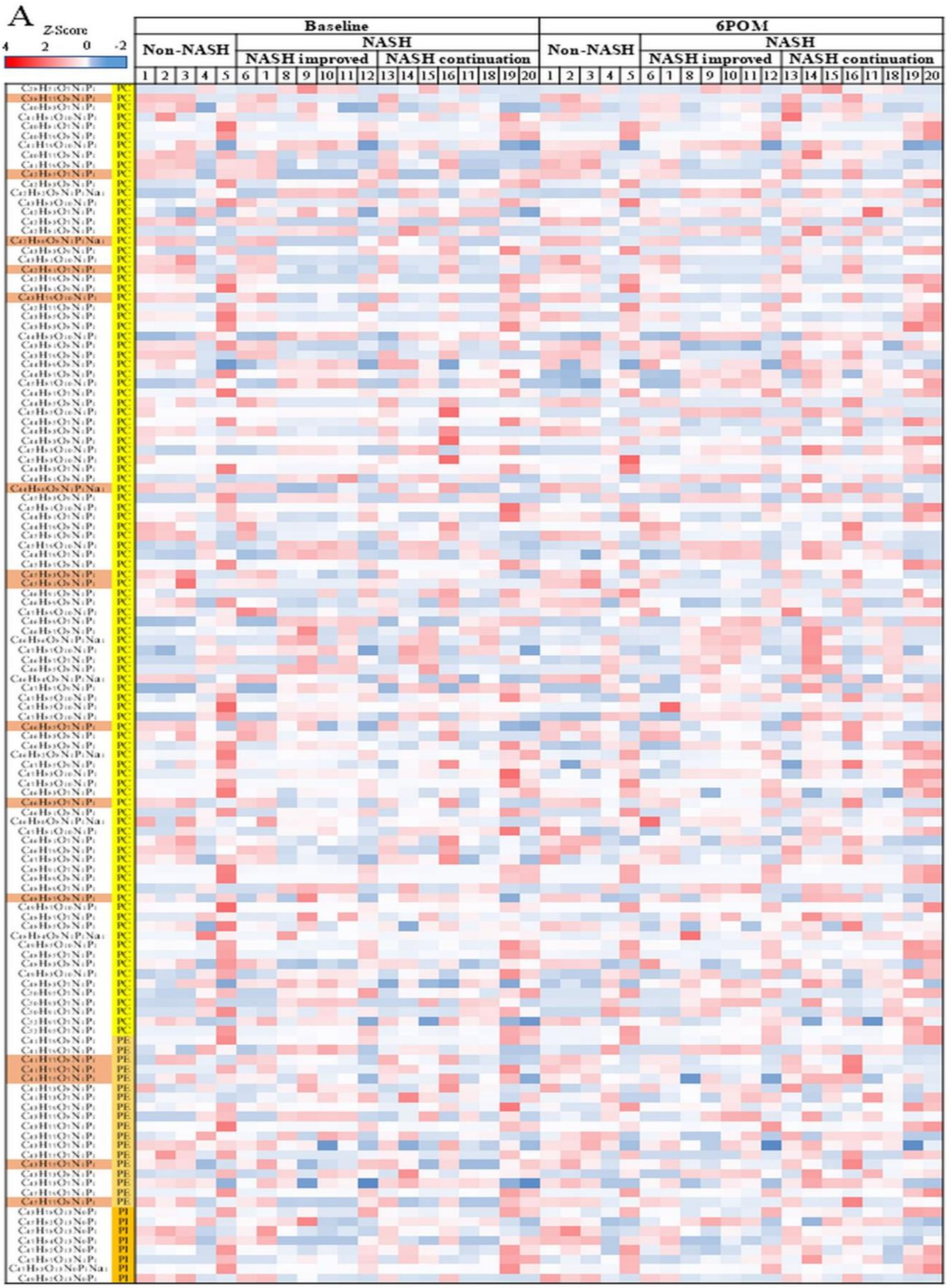
A
Z-Score
4 2 0 -2
Baseline
6POM
Non-NASH
NASH
NASH improved
NASH continuation
PC
PE
PI

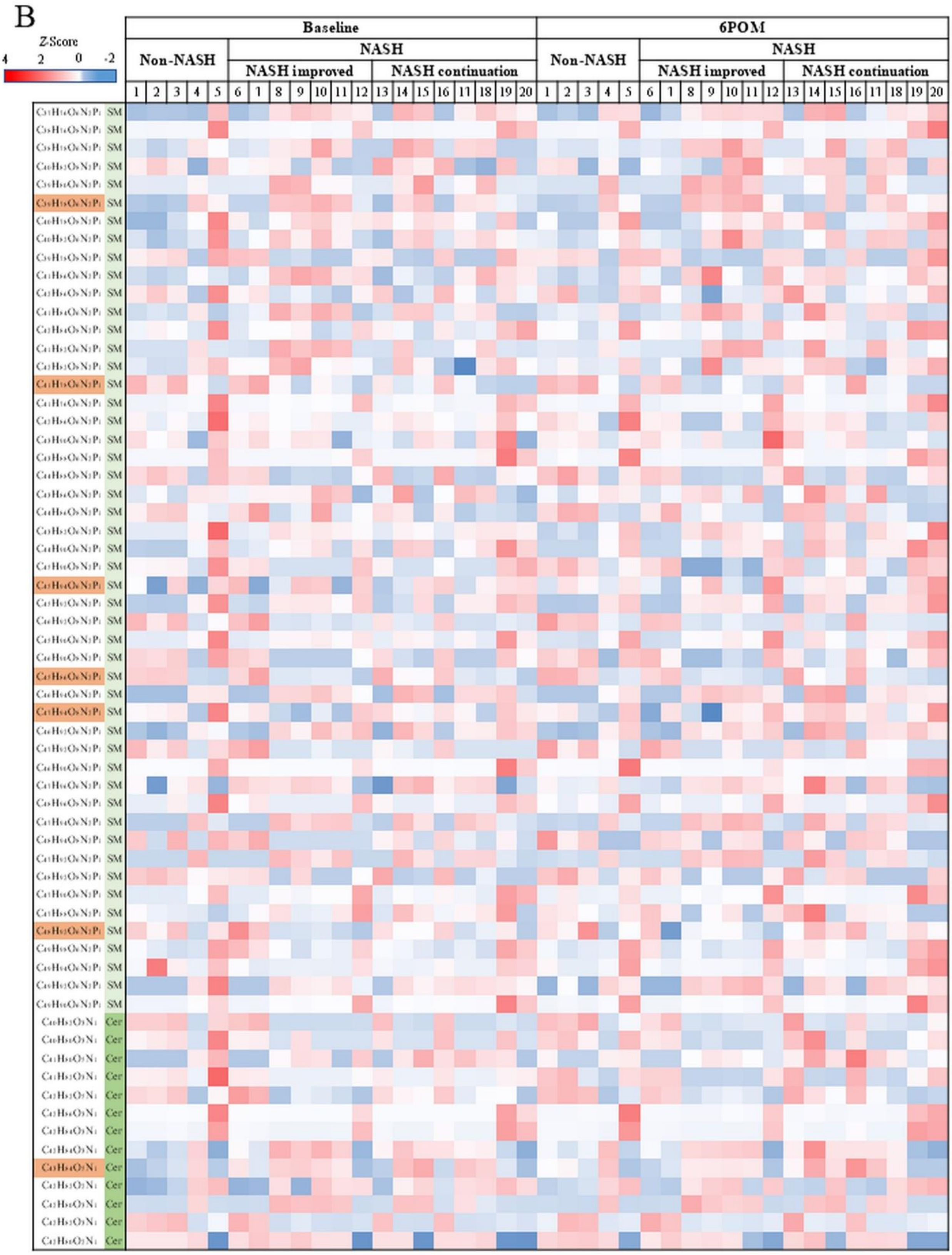

Figure S1. Heat map based on serum levels (Z-score) of 189 FFAs. (**A**) Heat map on phospholipids. (**B**) Heat map on sphingomyelin and ceramide.
